# Supplementary material for: Preparation of ESAT6-Fc Fusion Protein and Its Therapeutic Efficacy and Immune Mechanisms in Allergic Asthma Mice via Intranasal Immunization
Source: Molecules. 2025 Dec 19;31(1):7. doi: 10.3390/molecules31010007 (PMC12787016; doi:10.3390/molecules31010007)
Supplement: Supplementary file 1 [file molecules-31-00007-s001.zip › molecules-3972550-supplementary.pdf]

Supplementary Materials

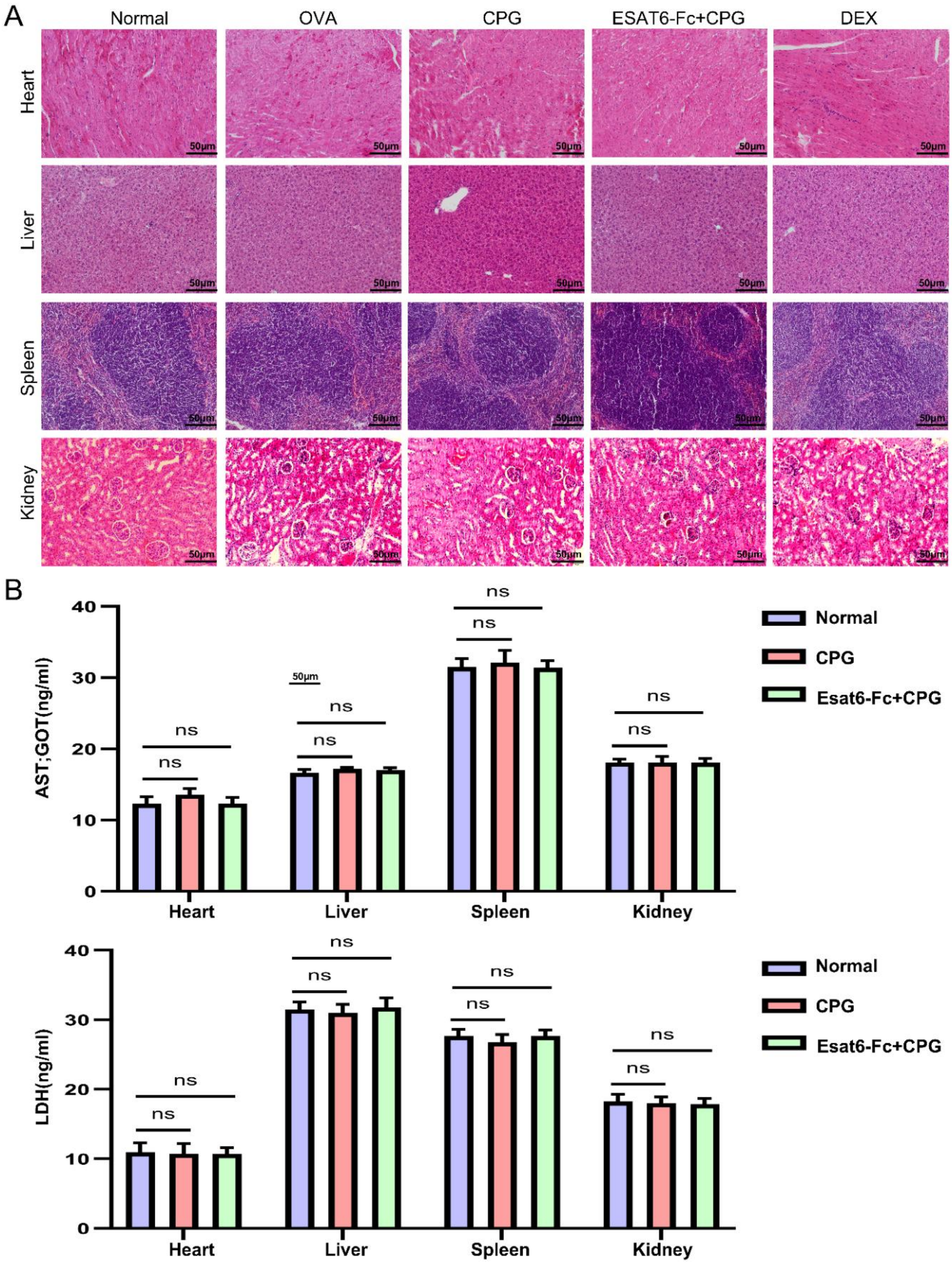

Figure S1. Assessment of pathological changes to the Heart, Liver, Spleen, and Kidney induced by ESAT6-Fc. (A) Assessment of pathological changes by H&E. (B) Assessment of pathological changes through the detection of AST and LDH in tissue homogenates.
